# Senotherapeutic-like effect of *Silybum marianum* flower extract revealed on human skin cells

**DOI:** 10.1371/journal.pone.0260545

**Published:** 2021-12-16

**Authors:** Jieun Woo, Seoungwoo Shin, Eunae Cho, Dehun Ryu, David Garandeau, Hanane Chajra, Mathilde Fréchet, Deokhoon Park, Eunsun Jung

**Affiliations:** 1 BioSpectrum Life Science Institute, Yongin-si, Gyeonggi-do, Republic of Korea; 2 Clariant Production, Toulouse, France; Lobachevsky University, RUSSIAN FEDERATION

## Abstract

Cellular senescence causes irreversible growth arrest of cells. Prolonged accumulation of senescent cells in tissues leads to increased detrimental effects due to senescence associated secretory phenotype (SASP). Recent findings suggest that elimination of senescent cells has a beneficial effect on organismal aging and lifespan. In this study, using a validated replicative senescent human dermal fibroblasts (HDFs) model, we showed that elimination of senescent cells is possible through the activation of an apoptotic mechanism. We have shown in this replicative senescence model, that cell senescence is associated with DNA damage and cell cycle arrest (p21, p53 markers). We have shown that *Silybum marianum* flower extract (SMFE) is a safe and selective senolytic agent targeting only senescent cells. The elimination of the cells is induced through the activation of apoptotic pathway confirmed by annexin V/propidium iodide and caspase-3/PARP staining. Moreover, SMFE suppresses the expression of SASP factors such as IL-6 and MMP-1 in senescent HDFs. In a co-culture model of senescent and young fibroblasts, we demonstrated that senescent cells impaired the proliferative capacities of young cells. Interestingly, when the co-culture is treated with SMFE, the cell proliferation rate of young cells is increased due to the decrease of the senescent burden. Moreover, we demonstrated *in vitro* that senescent fibroblasts trigger senescent process in normal keratinocytes through a paracrine effect. Indeed, the conditioned medium of senescent HDFs treated with SMFE reduced the level of senescence-associated beta-galactosidase (SA-β-Gal), p16^INK4A^ and SASP factors in keratinocytes compared with CM of senescent HDFs. These results indicate that SMFE can prevent premature aging due to senescence and even reprograms aged skin. Indeed, thanks to its senolytic and senomorphic properties SMFE is a candidate for anti-senescence strategies.

## 1. Introduction

Cellular senescence is a stress response induced by various stressors such as UV irradiation, pollution and replicative exhaustion. The Hayflick system of normal fibroblasts undergoing replicative senescence has been widely used in cellular senescence studies [1]. Senescence is characterized by morphological changes, high activity of senescence-associated beta-galactosidase (SA-β-gal), increase in senescence-associated secretory phenotype (SASP) characterized by cytokines and matrix metalloproteinase expression, reduction of lamin B1 expression and cytoplasmic translocation of nuclear HMGB1. These changes in senescent cells mediate aging and age related diseases in organisms such as Alzheimer disease, cognitive decline and osteoarthritis [2–7].

A fraction of senescent cells is increased with skin aging. Senescent fibroblasts constitute 20% to 60% of the dermis and release growth factors, including cytokines and degradative enzymes, which alter their microenvironment of extracellular matrix (ECM) and lead to skin aging. For example, matrix metalloproteinases secreted from senescent fibroblasts play a critical role in degradation of ECM [8]. Stromal-derived factor 1 (SDF1) deficiency in senescent fibroblasts also contributes to aging pigmentation by affecting the neighboring melanocytes [9]. Human epidermal keratinocytes (HEKs) exposed throughout their culture to factors secreted by senescent HDFs undergo post-senescence neoplastic emergence more frequently than unexposed cells [10]. The different works highlighted the importance of developing senotherapeutic approaches to selectively kill senescent cells (senolytics) or to suppress the senescence-associated secretory phenotype (SASP) driven inflammation associated with aging (senomorphics), in order to extend healthspan and potentially lifespan [11]. Moreover, these findings indicate that suppression of senescent fibroblasts (senolytic approach) or mitigation of their secretory profile (senomorphic approach) are relevant targets for anti-skin aging interventions.

Senolytic agents, which can selectively kill senescent cells, prevent and treat age-related diseases due to senescence process, and thereby prolong the healthy lifespan. Several compounds have been validated in animal models and have great potential for application in clinical trials. Senescent cells are resistant to apoptosis via up-regulation of pro-survival pathways that augment senescent cell burden. Senescent-cell anti-apoptotic pathways (SCAPs) are related to a network of BCL-2 family, PI3K/AKT, p53/p21/serpine, HIF-1α and HSP-90. Senolytic agents including dasatinib, quercetin [12], fisetin [13], FOXO4-interfering peptides [14], and Bcl-2 family inhibitors (ABT-263, ABT-737) [15–17] induce apoptosis in senescent cells through inhibition of SCAPs [18]. Recent studies have shown that natural compounds such as quercetin, fisetin, piperlongumine and the curcumin analogs are effective senolytic agents [19].

Senomorphics are typically understood as agents that suppress markers of senescence or their secretory phenotype without inducing apoptosis, providing a senotherapeutic effect by a mechanism different to that of senolytics [4, 5]. It has been shown that flavonoids such as quercetin and fisetin exert senomorphic properties [11, 20, 21].

In this study, we evaluated the potential of *Silybum marianum* flower extract (SMFE) as a senolytic and senomorphic agent in the treatment of skin aging but also for age-related diseases.

*Sylibum marianum*, commonly known as milk thistle is a plant belonging to the daisy family. Originally native of Southern Europe and Asia, it is now found throughout the world. The seeds of *S*. *marianum* have been used as herbal medicine in diseases of the liver and bile tract, due to their high concentration of bioactives such as silymarin, which exert anti-oxidant, anti-apoptotic, anti-inflammatory and anti-aging activity [22, 23]. However, the effect of *S*. *marianum* flower obtained without seeds on cellular senescence has yet to be investigated. Therefore, we investigated the impact of SMFE on senescence outbreak and on senescent HDF and keratinocytes behaviors. We demonstrate the ability of SMFE to limit senescence but also to eliminate remaining senescent cells by a senolytic and a senomorphic activity.

## 2. Materials and methods

### 2.1. Materials

Reagents were obtained as follows: ABT-737 was purchased from Abcam (MA, USA); 3-(4, 5-dimethyl-2-thiazolyl)-2, 5-diphenyltetrazolium bromide (MTT), dimethyl sulfoxide (DMSO) were ordered from Duchefa (Haarlem, The Netherlands). Hoechst 33342 and Dead Cell Apoptosis Kit were procured from Invitrogen (CA, USA), anti–γ-H2A.X, anti–p21^CIP1^, anti–cleaved caspase-3 and anti–cleaved PARP antibodies, Senescence SA β-Galactosidase Staining Kit were obtained from Cell Signaling Technologies (MA, USA). 2, 2-diphenyl-1-picrylhydrazyl (DPPH), L-ascorbic acid and transforming growth factor (TGF)-β human were purchased from Sigma-Aldrich (MO, USA).

### 2.2. Plant preparation and extraction

The flower of *S*. *marianum* was purchased from Dongjin farm (Jeonbuk, Korea). The powdered *S*. *marianum* flower was extracted with 70% ethanol. To prepare the ethanol extract, powdered *S*. *marianum* flower with seed removed (100 g) was extracted overnight with 70% (v/v) ethanol at 80°C for 3 hr. The supernatant was collected by filtration using a filter paper. Ethanol was removed via rotary vacuum evaporation (EYELA, Tokyo, Japan) then the extract was lyophilized.

### 2.3. Cell culture

Human neonatal dermal fibroblasts (HDFs; PCS-201-010™) were obtained from ATCC (VA, USA) and cultured in Dulbecco’s Modified Eagle’s Medium (DMEM; Welgene, Daegu, Korea) with 10% fetal bovine serum and penicillin/streptomycin (100 IU/50 μg/mL), whereas human epidermal keratinocytes derived from adult (HEKa; C0055C, Gibco, NY, USA) were grown and maintained in EpiLife™ medium in a humidified atmosphere containing 5% CO_2_ in air at 37°C. Young HDFs from passages 8 to 10 were used for the experiments. Replicative senescent HDFs were induced by long-term passaging of the cells in tissue culture. Cells acquired the senescence phenotype after 40 passages. Senescence induction by external stimulation was achieved by treating HDFs with 200 μM hydrogen peroxide, 100 nM doxorubicin, and 10 μM etoposide for 3 days.

### 2.4. Cell viability

Young and senescent HDFs were seeded at a density of 2 × 10^4^ cells on a 24-well plate and incubated with test materials for 3 days, followed by treatment with 0.1 mg/mL MTT solution for 3 h. The optical density was measured at 570 nm with a plate reader after the treatment.

### 2.5. SA-β-gal staining assay

HDF or HEKa cells were seeded at a density of 1 × 10^5^ cells in a 6-well plate, followed by incubation with test materials for 3 days. The cells were then stained using a Senescence β-Galactosidase staining kit (CST, MA, USA). SA-β-gal is used as a marker of senescent. The culture was incubated overnight after adding a staining solution; SA-β-gal in senescent cells stains blue. The proportion of senescent cells was determined as the number of senescent cells staining blue divided by the total number of cells. The average number of stained and total cells was determined for these ten fields (×100). For flow cytometry, HDF or HEKa cells were stained using a SPiDER-ßGal (Dojindo, Kumamoto, Japan). The cells were incubated for 15 min at 37°C after adding the staining solution. The cells were analyzed with a flow cytometer [24].

### 2.6. Enzyme-linked immunosorbent assay

The levels of MMP-1, IL-6 and procollagen type I in the cell supernatants were measured using the Human MMP-1 Quantikine ELISA Kit, Human IL-6 Quantikine ELISA Kit (R&D system Inc., MN, USA) and Procollagen Type I C-Peptide EIA Kit (Takara, Shiga, Japan) according to the manufacturer’s protocol. HDFs were treated with various concentrations of SMFE ranging from 100 to 200 μg/mL for 3 days and cell supernatants were collected.

### 2.7. Immunofluorescence staining

HDFs were seeded at a density of 2 × 10^4^ cells on a 24-well plate, followed by incubation with test materials for 3 days. HDFs were then fixed with 4% formaldehyde for 15 min at room temperature and blocked with 5% bovine serum albumin, 0.3% Tween 20 in PBS for 1 h. Anti–γ-H2A.X, anti–p21^CIP1^, anti–cleaved caspase-3, and anti–cleaved PARP antibodies (1:50 dilution; CST, MA, USA) was added to each well, followed by incubation overnight at 4°C. Cells were washed with PBS and incubated with Hoechst 33342 (1:10000; Invitrogen, CA, USA) for 10 min at room temperature. Fluorescence was subsequently analyzed via fluorescence microscopy (EVOS; Life Technologies GmbH, Darmstadt, Germany).

### 2.8. Annexin V/PI staining

HDF cells were seeded at a density of 1 × 10^5^ cells in a 6-well plate, followed by incubation with test materials for 3 days. After incubation, HDFs were harvested and washed once with PBS and resuspended with 100 μL 1X Annexin V binding buffer. Annexin V-Alexa Fluor 488 (Invitrogen, CA, USA) was added at a 1:20 dilution and PI was added at a final concentration of 1 μg/mL. The cells were stained for a further 15 min at room temperature and then diluted to a final volume of 400 μL, followed by analysis using a flow cytometer (Accuri C6; BD Biosciences, CA, USA). The fluorescence emission was measured at 530 nm and 575 nm with excitation at 488 nm.

### 2.9. Senolytic assay

The young and senescent HDFs were fluorescently labeled with PKH67 (green; Sigma-Aldrich, MO, USA) and PKH26 (red; Sigma-Aldrich, MO, USA), respectively. The cells were centrifuged at 400 g for 5 min and resuspended to the desired final concentration. HDFs were seeded on a 6-well plate (young: senescent = 1 × 10^4^ cells: 1 × 10^5^ cells). The mixture of senescent and young fibroblasts was then treated for 3 days with SMFE or ABT-737, a known senolytic drug, or untreated (control). HDFs were incubated for 3 days and then fixed with 4% formaldehyde for 15 min at room temperature. Cells were counterstained with Hoechst 33342 (nuclei, blue). Fluorescence was subsequently analyzed by fluorescence microscopy (EVOS; Life Technologies GmbH, Darmstadt, Germany). The percentage of cells positive for PKH26 (senescent, p40, red) and PKH67 (young, p8, green) was calculated relative to the total cell number.

### 2.10. Cell regeneration

HDFs were seeded on a 6-well plate (young: senescent = 1 × 10^4^ cells: 1 × 10^5^ cells). Following incubation with the test materials for 3 days, the cells were harvested and re-seeded (5 × 10^4^ cells) in a 6-well plate. After 3 days of incubation, the number of cells was counted.

### 2.11. Paracrine effects of senescent HDF on primary keratinocytes

HDFs were seeded at a density of 4 × 10^5^ cells on a 100 mm dish. After incubation with the test materials for 3 days, the medium was collected and centrifuged at 3000 rpm for 10 min to remove cell debris. The medium was then concentrated from the initial volume of 10 mL to a final volume of 1 mL using Vivaspin 20 centrifugal concentrator MWCO 3 kDa (Satorius, Goettingen, Germany). The amount of protein in the concentrated CM was detected using a BCA protein assay. The protein concentration of the prepared medium was adjusted to 3000 mg/mL for further experiments. The prepared medium was mixed in a ratio of 1:2 with keratinocyte growth medium for keratinocyte culture. The mixed medium was changed when the keratinocyte culture attained 50% confluency in the 6-well plate.

### 2.12. Quantitative polymerase chain reaction

The mRNA of fibroblasts or keratinocytes was isolated with TRIzol™ (Invitrogen, CA, USA) and chloroform (Sigma, MO, USA) according to the manufacturer’s manual. The isolated mRNA (1 mg) was reverse transcribed to cDNA with cDNA Synthesis Platinum Master Mix (GenDEPOT, TX, USA). Relative gene expression analysis was performed using 7500 Real Time PCR system (Applied Biosystems, CA, USA). Briefly, 20 μL of reaction mixture including each primer (Bioneer, Daejeon, Korea) and SYBR green PCR master mix (Enzo Life Sciences, NY, USA) was subjected to 40 cycles of RT-PCR (95°C for 15 s, 60°C for 60 s, and 95°C for 10 min). The comparative or ΔΔCt method was used for relative measurement of gene expression against GAPDH gene.

The following primers were used:

IL-1α: (Forward) TGTATGTGACTGCCCAAGATGAAG
    (Reverse) AGAGGAGGTTGGTCTCACTACC
IL-6: (Forward) AGACAGCCACTCACCTCTTCAG
    (Reverse) TTCTGCCAGTGCCTCTTTGCTG
MMP-1: (Forward) ATGAAGCAGCCCAGATGTGGAG
     (Reverse) TGGTCCACATCTGCTCTTGGCA
P16^INK4A^: (Forward) CTCGTGCTGATGCTACTGAGGA
     (Reverse) GGTCGGCGCAGTTGGGCTCC
P53: (Forward) CCTCAGCATCTTATCCGAGTGG
   (Reverse) TGGATGGTGGTACAGTCAGAGC
GAPDH: (Forward) CATCAAGAAGGTGGTGAAGCAGG
    (Reverse) AGTGGTCGTTGAGGGCAATGC

### 2.13. High performance liquid chromatography

The HPLC system used in this study was a Waters 2695 (Milford, MA, USA), equipped with Waters 2996 Photodiode Array (PDA) Detector. The Empower 2 software was used to control the analytical system and perform the data collection and processing. For HPLC-PDA was performed on a Phenomenex Synergi ^TM^ Hydro-RP (4.6 × 250 mm, 4 μm) column reversed-phase column protected by a C18 guard column from Phenomenex, Inc. (Torrance, CA, USA). The sample injection volume was 10 μL. The signal was monitored at 280 nm. The elution system used for the HPLC-PDA assay was a binary high-pressure gradient elution system with mobile phase A (0.1% TFA in H_2_O) and mobile phase B (acetonitrile). Elution gradient: 10% organic phase B, hold for 5 min; from 10 to 30% organic phase B in 25 min (linear gradient); from 30 to 50% organic phase B in 20 min (linear gradient); from 50 to 100% organic phase B in 10 min (linear gradient), hold for 10 min; then back to the starting condition in 1 min and re-equilibration for 9 min. The flow rate was 1.0 mL min^-1^. Each analysis required 80 min, including the re-equilibration time.

### 2.14. Measurement of DPPH radical scavenging activity

The DPPH radical scavenging activity of SMFE was determined by the modified previous method [25]. In brief, SMFE was mixed with 200 *μ*M DPPH reagent in equal amounts. After incubating this mixture for 30 min, the absorbance was measured at 517 nm using a spectrophotometer. DPPH radical scavenging activity was calculated by the following formula,

$$\%\;\text{scavenging}\;\text{activity}=\left[\left({\text{A}}_{\text{control}}-{\text{A}}_{\text{sample}}\right)/{\text{A}}_{\text{control}}\times 100\right].$$

A_control_ and A_sample_ indicate the absorbance of only DPPH reagent and SMFE -treated groups, respectively. L-ascorbic acid was used as a positive control with antioxidant activity.

### 2.15. Statistical analysis

All values were expressed as mean ± SD (standard deviation). All experiments were conducted in triplicate, and all data were evaluated on statistical significance by Student’s t-test using Statview software (Abacus Concepts, Piscataway, NJ, USA). p<0.05 was statistically significant between data.

## 3. Results

### 3.1. Validation of the replicative senescent model senescent cells

**Fig 1:**
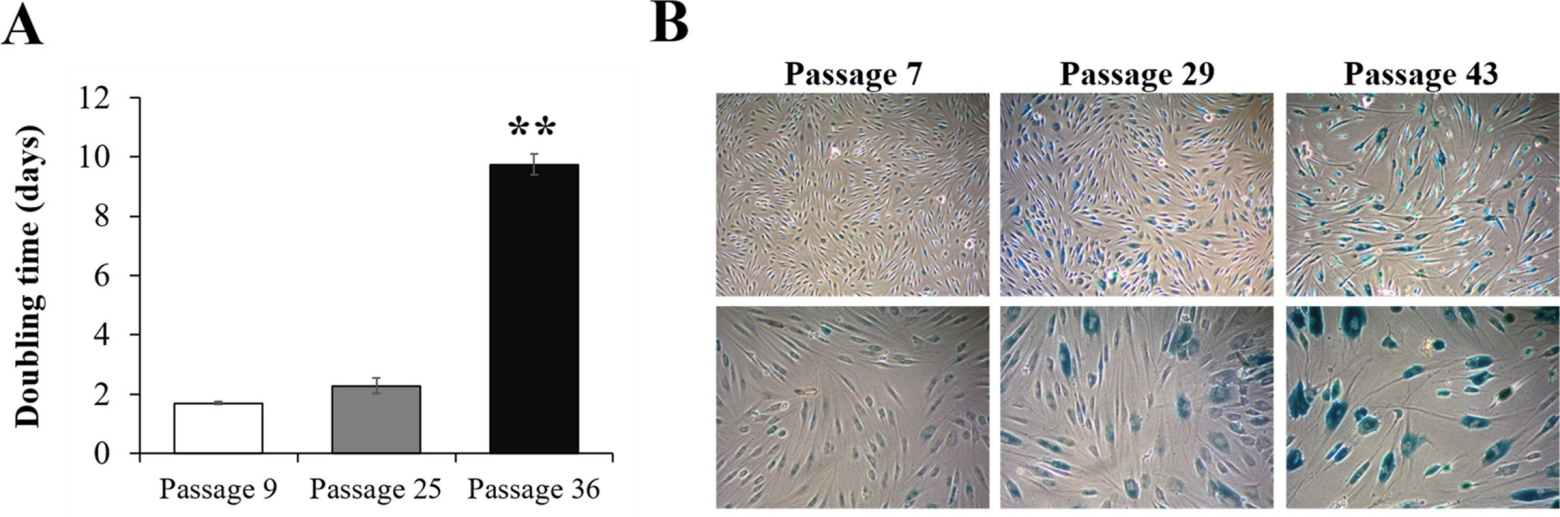
Establishment of senescence in fibroblast by successive passages in culture (replicative senescence model). (A) Calculation of the population doubling time and (B) Measurement of SA-β-gal activity according to the number of subcultures. Representative microphotographs are shown. Data are represented as mean ± SEM of three independent assays. **p < 0.01 compared to passage 9.

Several senescence pathways, both intrinsic and extrinsic, can stimulate the conversion of young cells into senescent cells. Increased expression of SA-β-gal is a known indicator of senescence. To induce replicative senescence, HDF cells were allowed to grow for more than 40 passages. In the case of fibroblasts, it has been reported that the doubling time increases sharply and cell proliferation decreases significantly when the passage is approximately 40 [26]. As shown in Fig 1A and 1B, compared to young fibroblasts (at passages 7~9), replicative senescent fibroblasts (at passages 36~43) showed a flattened cellular body, enlarged cytoplasm and reduced growth rate characterized by a significantly increased population doubling time (5-fold increase from 1.7 to 9.75 days) (Fig 1A). Additionally, SA-β-galactosidase staining was increased in the senescent fibroblasts (Fig 1B). Overall, these characteristics demonstrated the senescent phenotype of replicative senescent fibroblasts. These results validate the model and show its relevance to be used in all experiments presented in this paper.

### 3.2. SMFE repressed senescence mediated by DNA damage and activation of p53-p21^CIP1^ and p16^INK4A^

**Fig 2:**
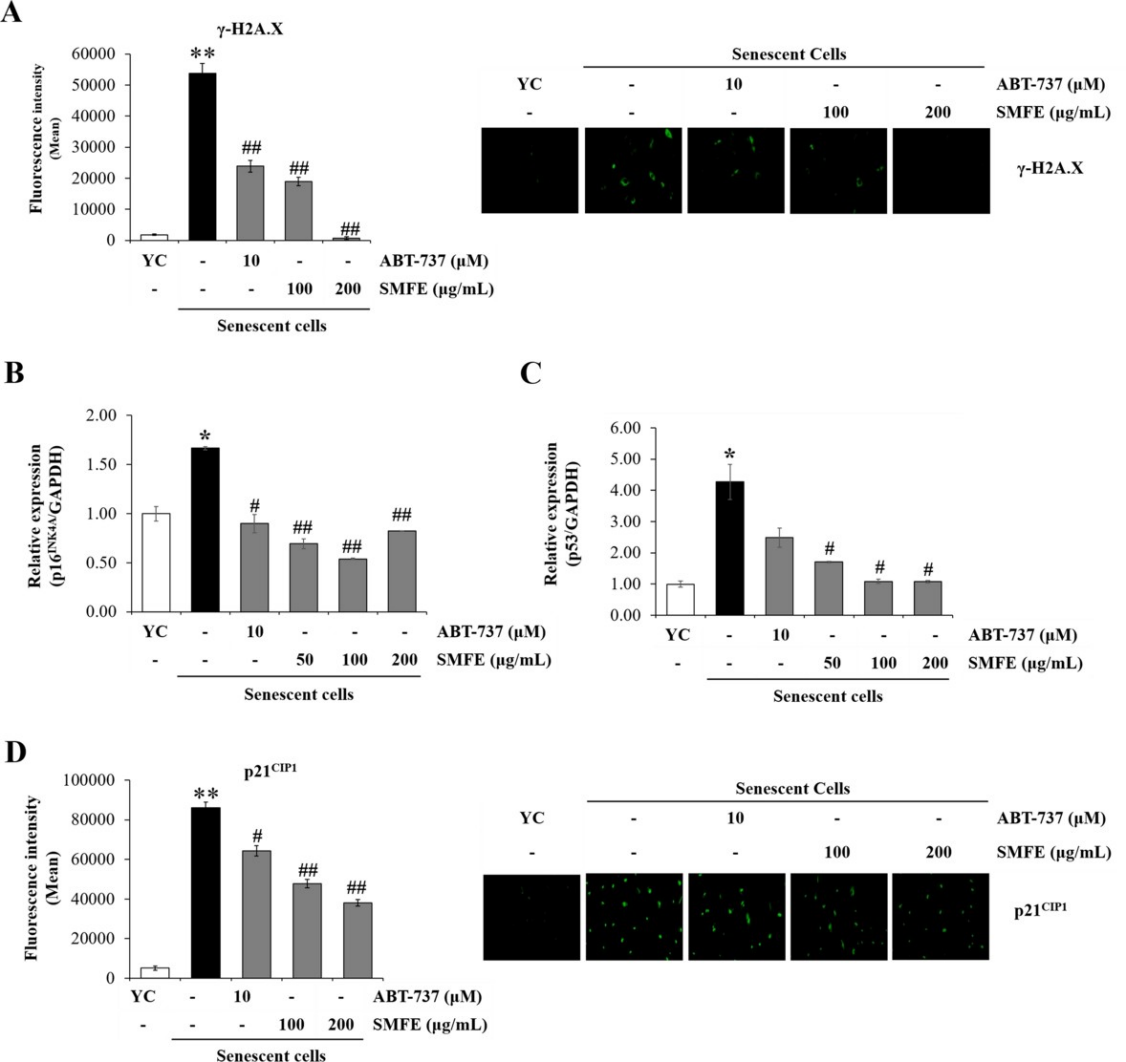
Inhibitory effect of SMFE against DNA damage marker and cell cycle markers. Senescent HDFs were treated with the indicated concentrations of extracts for 3 days. (A) Quantification of γ-H2AX staining intensity. (B, C) qRT–PCR for p16^INK4A^ and p53 mRNA expression. (D) Quantification of p21^CIP1^-staining intensity. Data are represented as mean ± SEM of three independent assays. *p < 0.05; **p < 0.01 compared to young cell control (YC), #p < 0.05; ##p < 0.01 compared to senescent cell control.

Recent studies have shown a causal relationship between DNA damage and senescence. The formation of DNA damage foci including γ-H2A.X (gamma-H2A.X) activated in uncapped telomere or persistent DNA strand breaks is now recognized as an indicator of DNA damage. Accordingly, γ-H2A.X staining is confirmed as a reliable quantitative indicator of DNA damage as well as senescence [27]. Accordingly, we analyzed the levels and activity of DNA damage via γ-H2A.X staining in SMFE-treated cells. As shown in Fig 2A, replicative senescent cells were positively stained for γ-H2A.X and the percentage of positive stained cells were further decreased using SMFE in a dose dependent manner. Taken together, these results suggest that SMFE reduces the formation of γ-H2A.X foci, and thereby attenuates the DNA damage, which triggers cellular senescence in replicative senescent cells.

The senescent state is also characterized by the activation of potent tumor suppressors p16^INK4A^ and/or p53. Since p16^INK4A^ appears to be exclusively expressed in senescent cells, the expression of p16^INK4A^ is the most reliable senescence marker [28]. Also, infliction of DNA damage in cells raises the expression of p53 rapidly including its post-translational modifications such as phosphorylation and acetylation. DNA damage induces p21^CIP1^ expression via p53. The levels of p21^CIP1^ are also a molecular hallmark of senescence and DNA-damage because they contribute to the induction of cellular senescence by inhibiting CDK2 and CDK4. We examined the expression of p53, p16^INK4A^ and p21^CIP1^ in SMFE-treated senescent cells. The results of RT-qPCR suggest that the expression of p53 and p16^INK4A^ was significantly decreased by SMFE treatment in senescent fibroblasts. In replicative senescent fibroblasts, the level of p53 and p16^INK4A^ increased significantly compared with that of young cells respectively 4.27-fold and 1.67-fold. Treatment with SMFE decreased strongly p53 and p16^INK4A^ expression (by nearly 1.07 and 0.54-fold) similar to young cells (Fig 2B and 2C). Further, we quantified the expression of p21^CIP1^ an established marker of senescence, using immunofluorescence. As expected, senescent cells showed a significant increase of p21^CIP1^ expression. SMFE reduced the level of p21^CIP1^ in a dose-dependent manner (Fig 2D).

These data suggest that SMFE-induced decrease in senescence mediated by DNA damage and activation of p53-p21^CIP1^ pathway as well as p16^INK4A^ in replicative senescent cells [29].

### 3.3. Senolytic effect of SMFE

**Fig 3:**
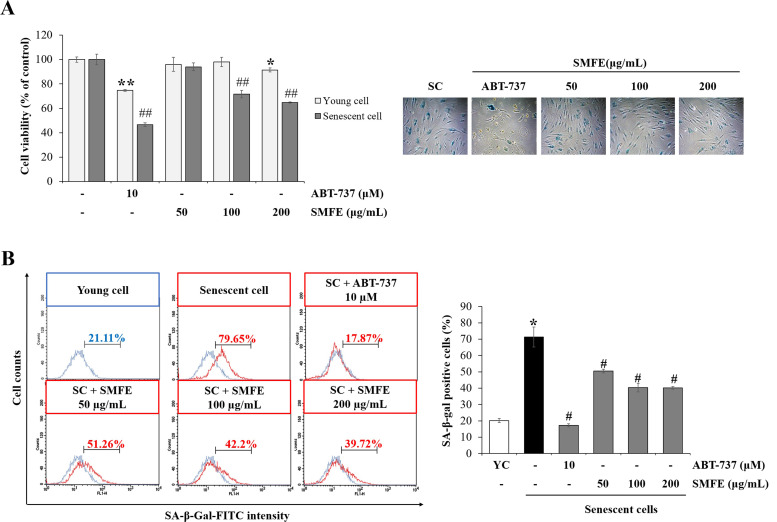
Senolytic effect of SMFE in a replicative senescence model. (A) Effect of SMFE on the viability of young cells (p8) and senescent cells (p40) after the cells were treated with the indicated concentrations of extracts for 3 days. (B) SA-β-gal activity in senescent cells. Representative flow cytometry plots are shown. Quantification of the percentage of positively stained cells (SA-β-gal FITC-positive) as in flow cytometry plots. Data are represented as mean ± SEM of three independent assays. *p < 0.05; **p < 0.01 compared to young cell control, #p < 0.05; ##p < 0.01 compared to senescent cell control. YC, young cell control; SC, senescent cell control.

To investigate the senolytic effect of SMFE on senescent fibroblasts, we determined the cell viability in senescent and young cells. When young cells (at passage 10) and senescent cells (at passage 40) were treated with SMFE for 72 h, SMFE selectively reduced in a dose dependent manner the viability of senescent cells to 71.7% at 100 μg/mL and 64.7% at 200 μg/mL, but did not reduce senescent cells at 50 μg/mL treatment and had no effect on the viability of young cells. These results show the selectivity of SMFE toward senescent cells. In contrast, ABT-737, known as a senolytic agent, reduced the viability of senescent cells by 74.7%, and young cells by 46.6% (Fig 3A). To determine the effect of SMFE on cellular senescence, SA-β-gal staining was performed. As shown in Fig 3B, SMFE decreased the beta-gal-positive cells of senescent HDFs. To normalize the effect of SA-β-gal staining according to the cell number, FACS analysis was performed additionally. The number of beta-gal-positive cells in senescent cells condition was significantly increased compared with young cells. However, SMFE decreased the SA-β-gal positive cells by 40.3% at 100 and 200 μg/mL. ABT-737 also reduced the SA-β-gal-positive cells by 17.2%. These results suggest that the senescent cells were selectively eliminated by SMFE treatment and as a result the proportion of senescent cells was decreased.

### 3.4. Induction of apoptosis by SMFE via caspase-3/PARP pathway

**Fig 4:**
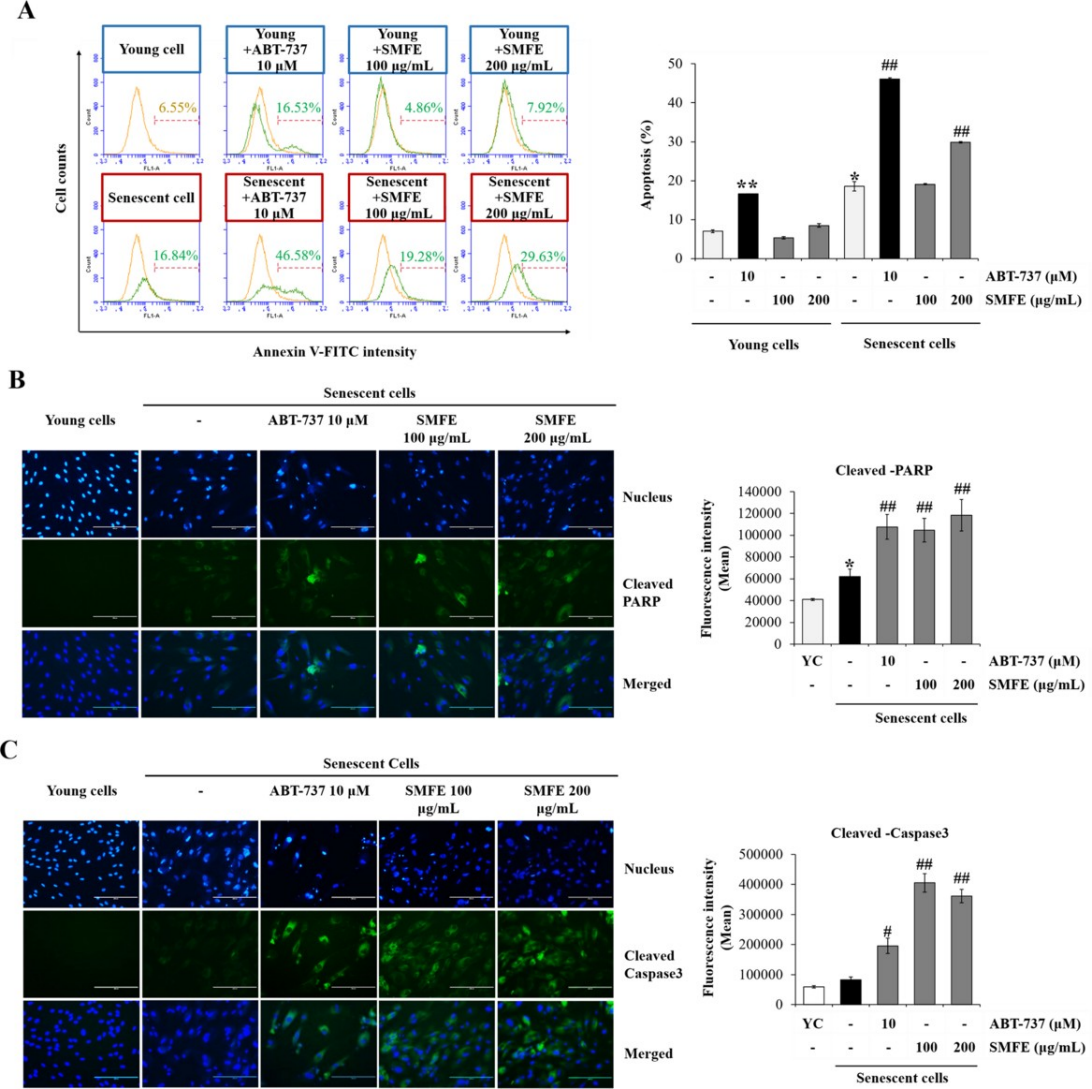
Induction of apoptosis by SMFE through caspase-3/PARP pathway. (A) Representative flow cytometry plots of apoptosis assay. Young and senescent cells were treated with vehicle or SMFE (100, 200 μg/mL) for 3 days. Cell apoptosis was assayed by flow cytometer after annexin V and PI staining. Quantification of the percentage of apoptotic cells (annexin V-FITC positive) 3 days after treatment as in (A) (right). (B-C) Representative immunofluorescence image and fluorescence intensity analysis of cleaved PARP (B) and cleaved caspase-3 (C) in young and senescent cells 3 days after incubation with vehicle or SMFE (100, 200 μg/mL). ABT-737 treated cells were used as a positive control. Data are represented as mean ± SEM of three independent assays. *p < 0.05; **p < 0.01 compared to young cell control, #p < 0.05; ##p < 0.01 compared to senescent cell control. YC, young cell control.

To investigate whether SMFE-induced decrease in the viability of senescent cells was mediated via apoptosis signaling, we determined the percentage of annexin V-FITC-positive apoptotic cells using FACS analysis. As shown in Fig 4A, SMFE did not induce apoptosis in young cells, whereas in senescent cells SMFE significantly increased the number and percentage of annexin V-FITC-positive apoptotic cells by 29.87% at 200 μg/mL compared with vehicle treatment (18.56%). ABT-737 induced apoptosis not only in senescent cells, but also in young cells.

To understand the apoptotic cell-death mechanism of SMFE, the caspase-3 and poly (ADP-ribose) polymerase (PARP) levels were analyzed using immunofluorescence staining assay. Indeed, apoptosis induction was associated with activation of caspase-3 and PARP cleavage [30]. As shown in Fig 4B and 4C, the levels of activated caspase-3 and the degradation of PARP were elevated in SMFE-treated senescent cells. This result showed that SMFE induced apoptosis of senescent cells via caspase 3/PARP pathway.

### 3.5. Effect of SMFE on removal of senescent cells and cell proliferation

**Fig 5:**
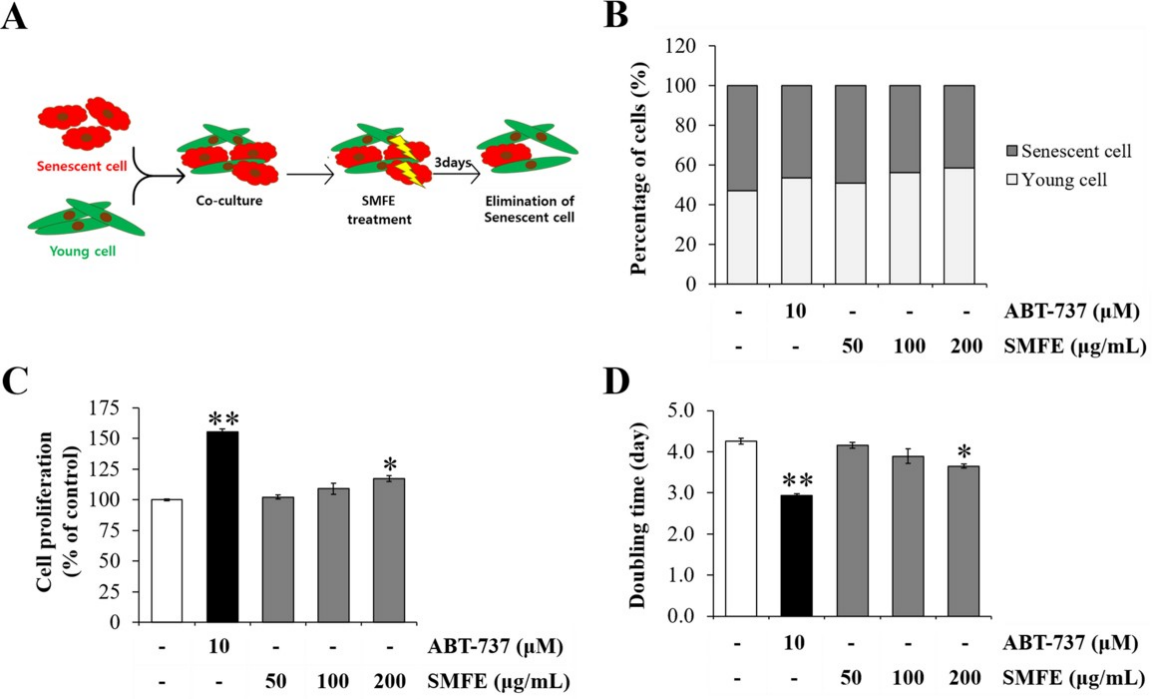
Effect of SMFE on removal of senescent cells and cell proliferation. (A) Schematic representation of co-culture process (B) Rate of young and senescent cells after SMFE treatment for 3 days. PKH26 (senescent, p40, red)-positive cells and PKH67 (young, p8, green) -positive cells were calculated the percentage compared to the total cell number. (C-D) The young and senescent cells were mixed 1:9, and treated with the SMFE for 3 days, then the cells harvested and re-seeded. After 3 days of incubation, cell growth rate and population doubling time measured by cell counting (C) Cell proliferation analyzed after 6 days of incubation of re-seeding cells. (D) The population doubling time analysis. Data are represented as mean ± SEM of three independent assays. *p < 0.05; **p < 0.01 compared to the vehicle.

Until now, we established that SMFE selectively kills senescent cells, thereby reducing the proportion of senescent cells among all cells. The results now confirm the senolytic effect of SMFE on senescent cells co-cultured with young cells. We investigated whether SMFE increased the cell growth via removal of senescent cells. We designed a co-culture experiment (Fig 5A) based on previous reports [31]. HDFs at early passages (p8, young cells) were labeled with PKH67 in green and the corresponding cells at late passages (p40, senescent cells) were labeled with PKH26 in red. The young and senescent cells were mixed in 1:9 ratio and treated with SMFE, followed by fluorescence microscopy. Since the proliferation of senescent cells was significantly lower than that of young cells, the ratio of senescent cells was larger than that of young cells. As shown in Figs 5B and S1, the percentage of senescent cells was decreased while that of young cells increased in the SMFE-treated group compared with the vehicle group. To determine whether the elimination of senescent cells increased the cell growth rate, the treated cells were harvested and re-seeded without sample treatment. As shown in Fig 5C and 5D, the cell proliferation was increased in the SMFE-treated group in previous passages. Cell doubling time was also reduced in the SMFE-treated group compared with the control group. These results were attributed to the increased proportion of young cells based on the senolytic effect of SMFE and ABT-737.

### 3.6. SMFE shows a senomorphic effect through the inhibition of Senescence-associated secretory phenotype (SASP) production by senescent fibroblasts

**Fig 6:**
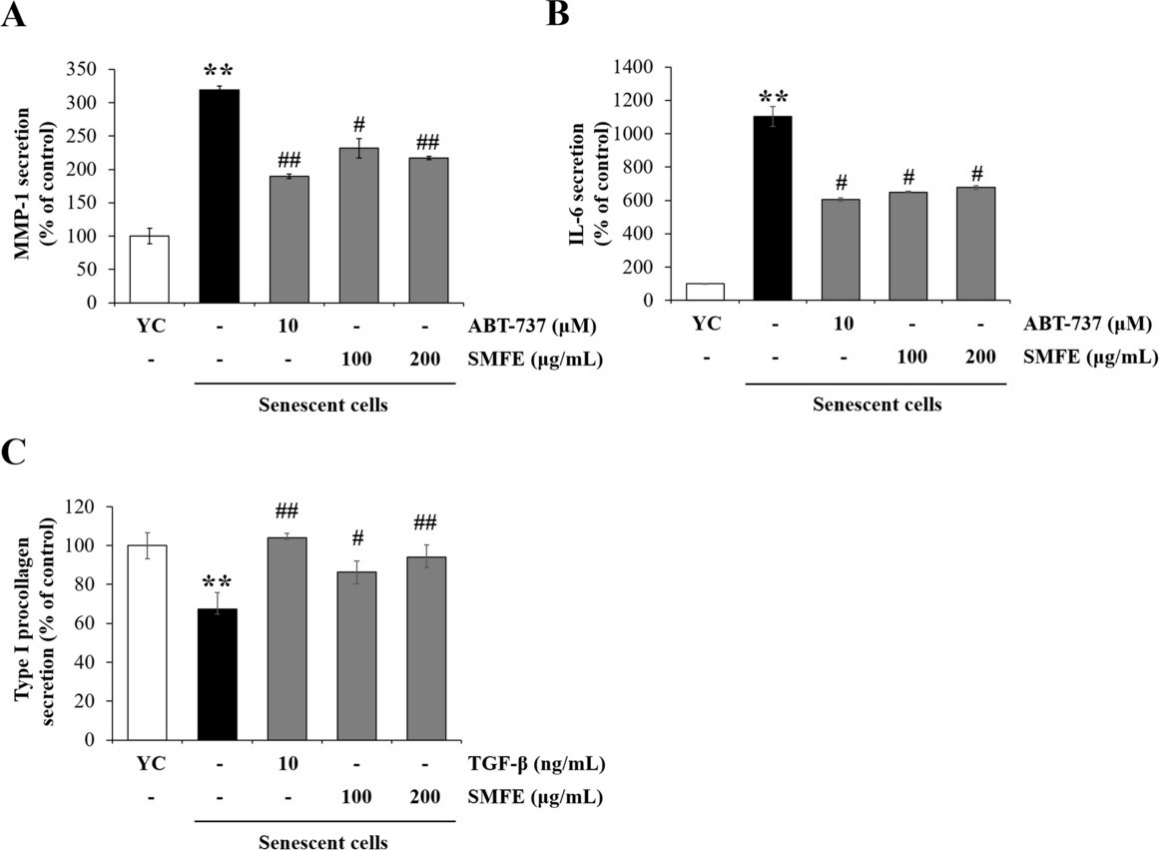
Effect of SMFE on SASPs and type-1 procollagen expression in senescent cells. Senescent HDFs were treated with the indicated concentrations of extracts for 3 days. The secretion of MMP-1 (A), IL-6 (B) and collagen production (C) in the culture supernatant was measured using an ELISA kit. Data are represented as mean ± SEM of three independent assays. **p < 0.01 compared to young cell control, #p < 0.05; ##p < 0.01 compared to senescent cell control.

It is widely known that the expression and secretion of inflammatory cytokines and metalloproteinases are increased in senescent cells. SASP contributes to local and systemic low-grade inflammation during aging, age-related degenerative phenotypes, and impaired physical function. Therefore, we measured the secreted levels of IL-6 and MMP-1 using ELISA assay. Compared to young cells, the secretions of IL-6 and MMP-1 were significantly increased in senescent cells. SMFE at 100 μg/mL reduced the level of IL-6 and MMP-1 secretion respectively about 59% and 73%, compared to senescent control. (Fig 6A and 6B). MMP-1 is also a major enzyme that initiates the breakdown of collagen and extracellular matrix (ECM) proteins in human skin, and type I collagen fibrils are the major structural components of the dermal ECM [32]. We found that type I procollagen protein levels decreased by 67.3% in senescent cells compared to young cells. SMFE treatment significantly increased type I procollagen protein levels to a maximum level of 140% at a concentration of 200 μg/mL, compared to senescent control (Fig 6C). We used TGF-β as a positive control for collagen production [33]. This is expected to be related to the results of suppression of MMP-1 expression by SMFE treatment in senescent dermal fibroblasts.

Taken together, SMFE can be considered a senomorphic ingredient through mitigating effect on SASP of senescent cells.

### 3.7. Effect of senescent HDFs following SMFE treatment of keratinocytes

**Fig 7:**
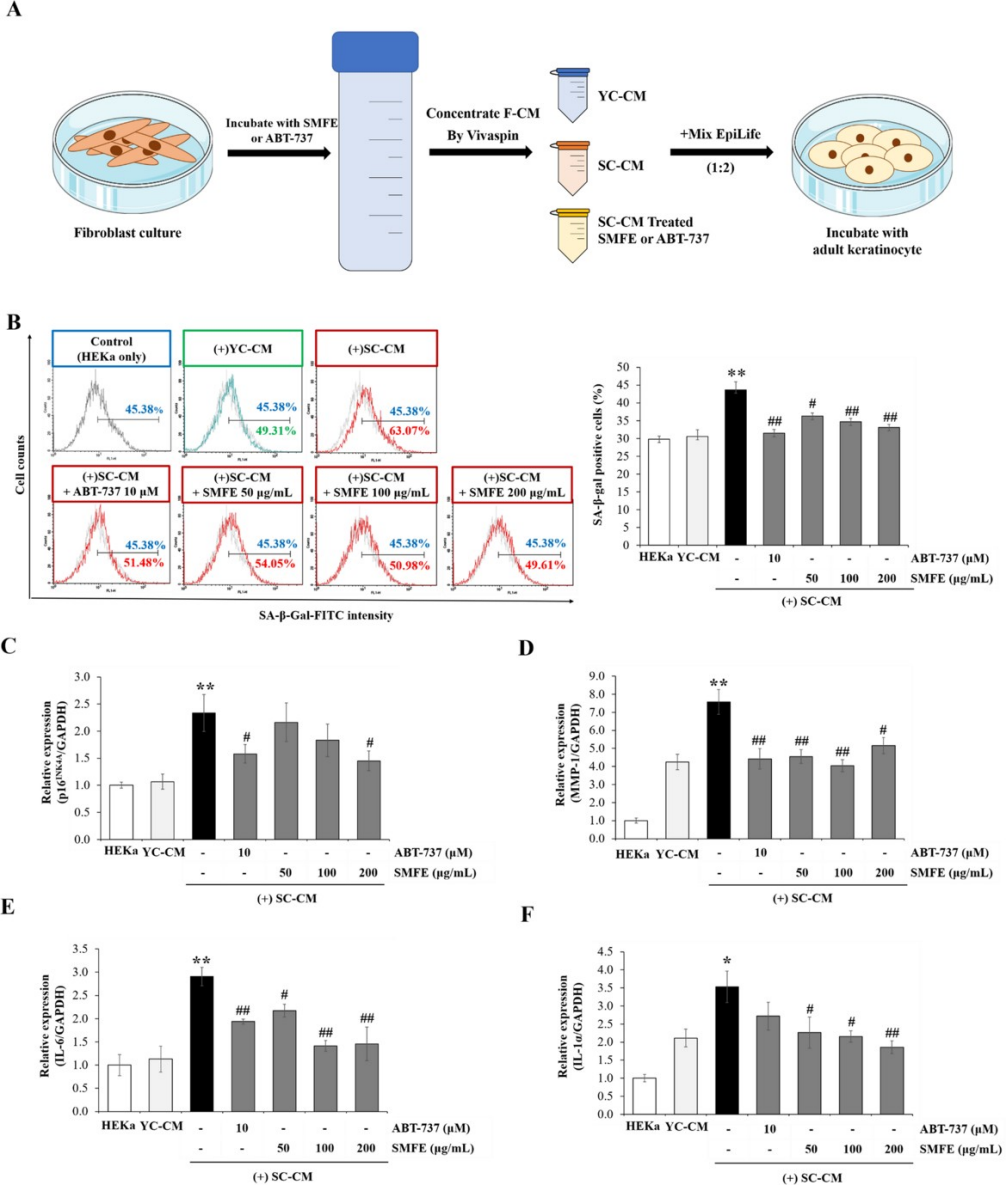
Effect of F-CM with or without SMFE on adult keratinocytes senescence. (A) Process for the preparation of concentrated F-CM. (B) SA-β-gal activity in senescent cells. Representative flow cytometry plots are shown. Quantification of the percentage of positively stained cells (SA-β-gal FITC-positive) as in flow cytometry plots. (C) qRT–PCR for p16^INK4A^ mRNA expression. (D-F) qRT–PCR for MMP-1, IL-6, and IL-1α mRNA expression. Data are represented as mean ± SEM of three independent assays. *p < 0.05; **p < 0.01 compared to young cell control, #p < 0.05; ##p < 0.01 compared to senescent cell control. YC, young cell control; SC, senescent cell control; CM, conditioned medium.

It’s well described that keratinocytes exert a crucial regulatory role on fibroblast functions such as proliferation and migration [34]. Two properties crucial for the maintenance of skin homeostasis and repair. Thus, it appears clear that protecting keratinocytes can be a way to prevent premature aging. In our study, we demonstrate that senescent fibroblasts negatively modulate the function of normal keratinocytes. Indeed, we have shown that keratinocytes treated with CM of senescent HDF cells (SC-CM) showed a strong increase (43.2%) in the number of SA-β-gal-positive cells. However, treatment with CM of senescent HDFs exposed to SMFE (SMFE-CM) decreased SA-β-gal-positive cells to 36.29% (50 μg/mL) and to a maximum level of 33.15% (200 μg/mL) compared with SC-CM (Fig 7B). When assessing the mRNA level of SASP by qRT-PCR, the 2.33-fold increase in p16^INK4A^ gene expression of keratinocytes by SC-CM was significantly reduced to 2.16-, 1.83-, and 1.45-fold at 50, 100, and 200 μg/mL SMFE concentrations, respectively (Fig 7C). In addition, we measured the gene expression of MMP-1, IL-6 and IL-1α on keratinocytes treated with CM (of senescent HDF). The gene expression of MMP-1, IL-6 and IL-1α was increased in keratinocytes treated with SC-CM. However, the SMFE-CM-treated group showed a significant decrease in MMP-1, IL-6 and IL-1α expression in keratinocytes compared with the SC-CM treated group (Fig 7D–7F). These data suggest that SMFE can alleviate the negative effects of senescent HDF on neighboring keratinocytes.

### 3.8. Phytochemical characterization of *Silybum marianum* flower extract (SMFE)

**Fig 8:**
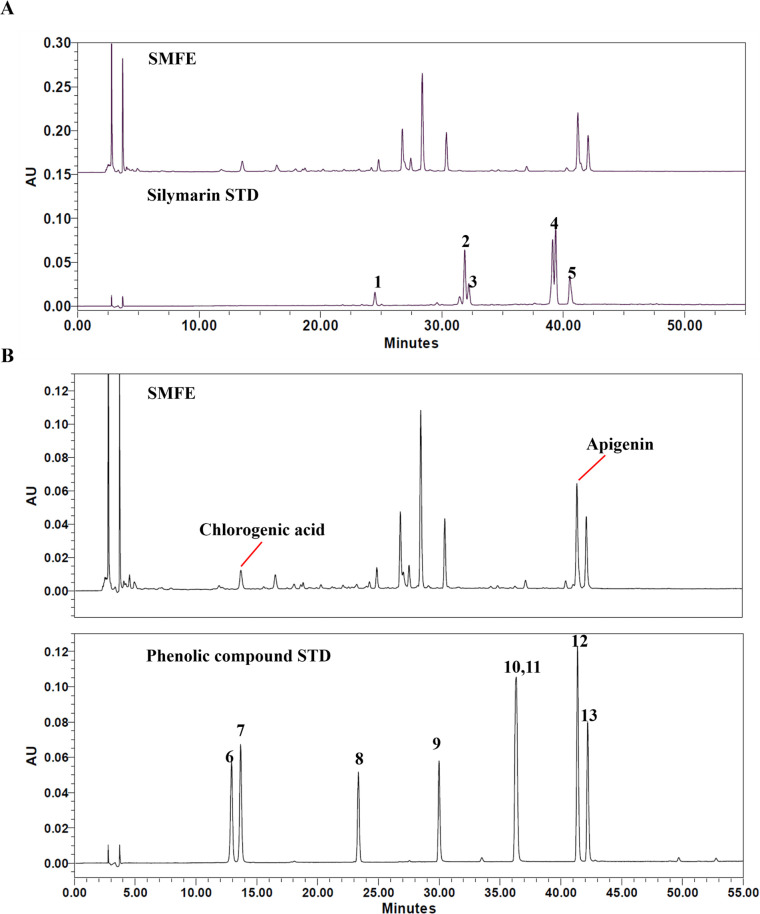
HPLC chromatogram of SMFE at 290 nm. (A) Silymarin analysis of SMFE. (B) Phenolic compound analysis of SMFE. 1, Taxifolin; 2,Silychristin; 3, Silydianin; 4,Silybinin A,B; 5, Isosilybin; 6, Scopolin; 7, chlorogenic acid; 8, Scopoletin; 9, Myricetin; 10, Quercetin; 11, Luteolin; 12, Apigenin; 13, Kaempferol.

Silymarin is the most well-known active ingredient in *S*. *marianum*. It is an isomeric mixture of flavonoid complexes. The main polyphenolic component of silymarin is silibin (approximately 50–70%) that is also known to exhibit the highest biological activity. Other silymarin components include silychristin (about 20%), silydianin (about 10%), isosilybin, and their flavonoid precursor, taxifolin [35]. As shown in Fig 8A, we did not detect any silymarin peak in SMFE, indicating that the flower does not contain the silymarin component. We also analyzed the phenolic compounds including scopolin, chlorogenic acid, scopoletin, myricetin, quercetin, luteolin, apigenin, and kaempferol. As shown in Fig 8B, the major peak, tR of 41.3 min, was identified as apigenin. Its content was calculated as over 0.24% (w/w).

## 4. Discussion

Cellular senescence is accompanied by high levels of p16^INK4a^ and/or p21^CIP1^, which induce cell cycle arrest, high activity of SA-β-gal, increased SASP and resistance to apoptosis. Moreover, replicative senescence is induced by DNA damage (characterized by the expression of γ -H2A.X) due to the combination of continued proliferation and an increased resistance to apoptosis. This results in the accumulation of senescent cells, which contributes to age-related diseases and carcinogenesis [13]. In recent years, anti-aging studies targeted not only the suppression of cellular senescence, but also the effective elimination of senescent cells. The results of this study showed that SMFE decreased the DNA damage-mediated cellular senescence (reduced level of γ -H2A.X) and induced selective elimination of senescent cells through an apoptotic mechanism. Indeed, SMFE induced selective toxicity against senescent but not young cells. The results of annexin V staining demonstrate that only senescent cells treated with SMFE were annexin V-FITC-positive cells. SMFE induced only in senescent cells the upregulation of the apoptotic proteins, cleaved caspase-3 and PARP (Fig 4). Based on the work of Yosef et al., we postulate that reducing senescent cell burden of a tissue can lead to a decreased inflammation and increased capacity of renewal, which resulted in recovery of cellular function. Indeed, they have shown that fisetin, a known senolytic substance improves regenerative capacity, through targeting p21 aberrant expression and reduced the tissue expression of p16 ^INK4a^ and p21 ^CIP1^ [36, 37]. In our co-culture experiments of senescent cells and young cells, we demonstrate that SMFE enhanced cell proliferation of young cells via a senolytic activity (Figs 5 and S1).These findings suggest that the cellular toxicity of SMFE against senescent fibroblasts was mediated by apoptosis via caspase-3/PARP signaling pathways, which contribute to restoration of cell proliferation in the co-culture model (Figs 4 and 5) [38]. This effect was confirmed by the effect of SMFE on the levels of p53-p21^CIP1^ and p16^INK4A^ known as representative senescence markers.

Besides replicative senescence, senolytic drugs can be used to treat senescence induced by variety of stresses including oxidative stress, anti-cancers drugs (etoposide and doxorubicin) and carcinogens. Senescence can be efficiently induced in cultured cells by DNA-damaging drugs such as doxorubicin and etoposide. A plethora of anti-cancer drugs have been shown to promote a form of therapy-induced senescence both *in vitro* and *in vivo* [39]. Treatment with senolytic drug navitoclax, ABT-263 and ABT-737, eliminates persisting senescent tumor cells following exposure to etoposide or doxorubicin chemotherapy. SMFE also showed senolytic activity in these stress induced senescent cells models (Figs 3A and S2).

Recently senomorphics, which inhibit SASP from senescent cells, has been reported as an anti-aging strategy [40]. The SASP produced by senescent cells has a detrimental effect on microenvironment. Chronic exposure to SASP reinforces cell-cycle arrest in a paracrine manner, which potentially leads to senescent phenotype of neighboring cells and abnormal regeneration of the surrounding tissue. In this study, SMFE inhibited the production of SASP such as IL-6 and MMP-1 in senescent fibroblasts. In particular, at low concentrations, only senomorphic effects were shown without senolytic effects (S3 Fig). Inhibitors of IκB kinase (IKK) and nuclear factor (NF) - κB [41], free radical scavengers [42], mTOR inhibitors and Janus kinase (JAK) pathway inhibitors have been reported to show senomorphic ability [40]. Free-radical scavenging activity of SMFE can be involved in inhibitory activity on SASP production (S4 Fig). But further study is needed to determine the effect of SMFE on mTOR, (NF)-κB and JAK pathways to elucidate the pathway involved in senomorphic activity. The SASP produced from senescent fibroblasts also affects the proliferation, differentiation, and stress responses of keratinocytes [43, 44]. In this study, we found that keratinocytes treated with CM of senescent HDF cells (SC-CM) showed an increase in the number of SA-β-gal-positive cells and MMP-1, IL-6 and IL-1α. These findings suggest that SASP secreted by senescent fibroblasts has detrimental effects on keratinocytes by inducing cellular senescence and inflammation.

Instead, CM of senescent HDFs treated with SMFE (SMFE-CM) decreased the SA-β-gal expression and the expression of MMP-1, IL-6 and IL-1α in keratinocytes compared with the SC-CM treated group (Fig 7). Alleviating negative effects of SMFE on keratinocytes may depend on the senomorphic activity in fibroblasts. In addition, we cannot exclude the possibility that senolytic activity of SMFE may contribute to the decreased SASP in SMFE-CM.

Silymarin is a representative component present in milk thistle fruits and seeds. It has been widely known to induce antioxidant and hepatoprotective activities. Silibinin, a major active constituent of silymarin, was reported to induce growth inhibition and apoptosis in tumor cells and to potentiate the effects of doxorubicin [45, 46]. These results suggest that silymarin may exert senolytic effect, but SMFE did not contain the silymarin component. Instead, flavonoids such as apigenin and chlorogenic acid were detected. The major peak identified was apigenin, which is a natural flavonoid with anti-proliferative and apoptotic activity in various types of tumor cells. Previous studies have reported that apigenin inhibits the expression of various anti-apoptotic Bcl-2 family proteins, including Bcl-xL, Mcl-1 and Bcl-2 in several cancer cell lines [47]. Further, the levels SASP was suppressed by apigenin through inhibition of IL-1α signal pathway in senescent fibroblasts [48]. These findings suggest that flavonoids such as apigenin present in SMFE may exert senolytic but also senomorphic activities in senescent fibroblasts.

In additional tests, we found that apigenin showed senolytic effect. However it cannot be considered as major compound for senolytic activity, because its effective concentration was much higher than the concentration that can be judged as an effective compound based on content of apigenin in extract. From these results, we can interpret that there are other active compounds which can exert superior senolytic activity than apigenin or show synergistic activity with apigenin in SMFE. The identification of active compound and its reaction with apigenin on senolytic activity need to be confirmed in further study.

Since several studies reported that synthetic senolytic chemical show side effect such as toxicity to normal cell despite the low effective concentration, the demand for natural substances that can offer similar effectiveness with fewer side effects is increasing [49]. Plant-derived compounds such as olive polyphenols, green tea catechins, resveratrol, fisetin has been reported to exert senolytic or senomorphic activity [50]. The effective concentration of these active compounds is less than 10 μg/mL while that of SMFE is 100–200 μg/mL. The effective concentration of SMFE *in vitro* is too high for *in vivo* application, and there is no indicator substance for pharmacokinetic studies. Therefore, it is necessary to trace and identify the active compound for senolytic effect in SMFE rather than directly applying the extract to senolytic drug and then examine its potential as a drug.

Altogether, these results demonstrate the anti-aging characteristics of SMFE. Indeed, we demonstrated that SMFE mitigates the senescence phenotype in a senescent model of HDF and thereby promotes the elimination of these deleterious cells via a selective senolytic activity. Our data confirmed also that this dual senotherapeutic approach “senolytic and senomorphic” is possible. Indeed, SMFE acts simultaneously as a senolytic agent, and a senomorphic agent (SASP modulator as well as a cell growth promoter, and reprogram senescent toward a metabolic phenotype similar to young cells), thereby indicating its multi-faceted attributes that can be used to develop anti-aging or age-delaying therapies. Therefore, SMFE can be proposed as a substance regulating senescence and degenerative diseases involving the accumulation of senescent cells.

## Supporting information

S1 Fig Senolytic effect of SMFE in replicative senescence model. Representative immunofluorescence image and fluorescence intensity analysis (upper panel). Exemplary phase-contrast and fluorescence microscopic images of young cells (PKH67, green, p8) and senescent cells (PKH26, red, passage40), (lower panel) senescent cells and cell nuclei (Hochest 33342, blue) overlap of images.
(TIF)

S2 Fig Senolytic effect of SMFE in senescence model caused by external stimulation, hydrogen peroxide (A), etoposide (B), and doxorubicin (C). Data are represented as mean ± SEM of three independent assays. *p < 0.05; **p < 0.01 compared to young cell control, #p < 0.05; ##p < 0.01 compared to senescent cell control. SC, senescent cell control.
(TIF)

S3 Fig Senolytic effect and SASP inhibitory effect of SMFE at lower concentration in replicative senescence model. (A) Effect of SMFE on the viability of young cells (p8) and senescent cells (p40) after the cells were treated with the indicated concentrations of extracts for 3 days. The secretion of MMP-1 (B) and IL-6 (C) in the senescent cell culture supernatant was measured using an ELISA kit. *p < 0.05; **p < 0.01 compared to young cell control, #p < 0.05; ##p < 0.01 compared to senescent cell control.
(TIF)

S4 Fig DPPH free radical scavenging effect of SMFE. Data are represented as mean ± SEM of three independent assays. *p < 0.05; **p < 0.01 compared to control.
(TIF)

S1 Data (XLSX)
