# Supplementary figures and images for: Senotherapeutic-like effect of Silybum marianum flower extract revealed on human skin cells
Source: PLoS One. 2021 Dec 16;16(12):e0260545. doi: 10.1371/journal.pone.0260545 (PMC8675675; doi:10.1371/journal.pone.0260545)

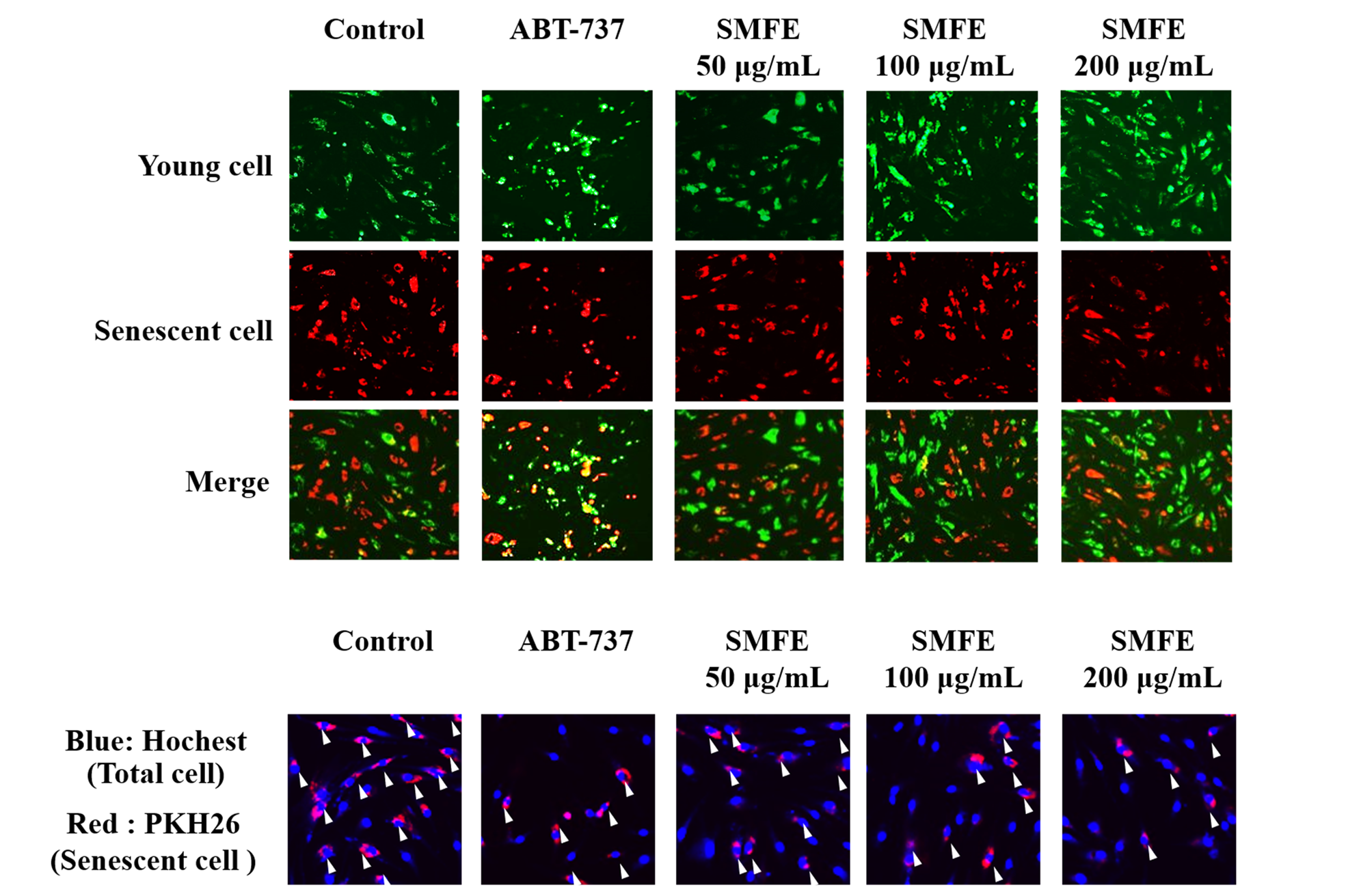

Supplement: S1 Fig — Representative immunofluorescence image and fluorescence intensity analysis (upper panel). Exemplary phase-contrast and fluorescence microscopic images of young cells (PKH67, green, p8) and senescent cells (PKH26, red, passage40), (lower panel) senescent cells and cell nuclei (Hochest 33342, blue) overlap of images. (TIF) [file pone.0260545.s001.tif]

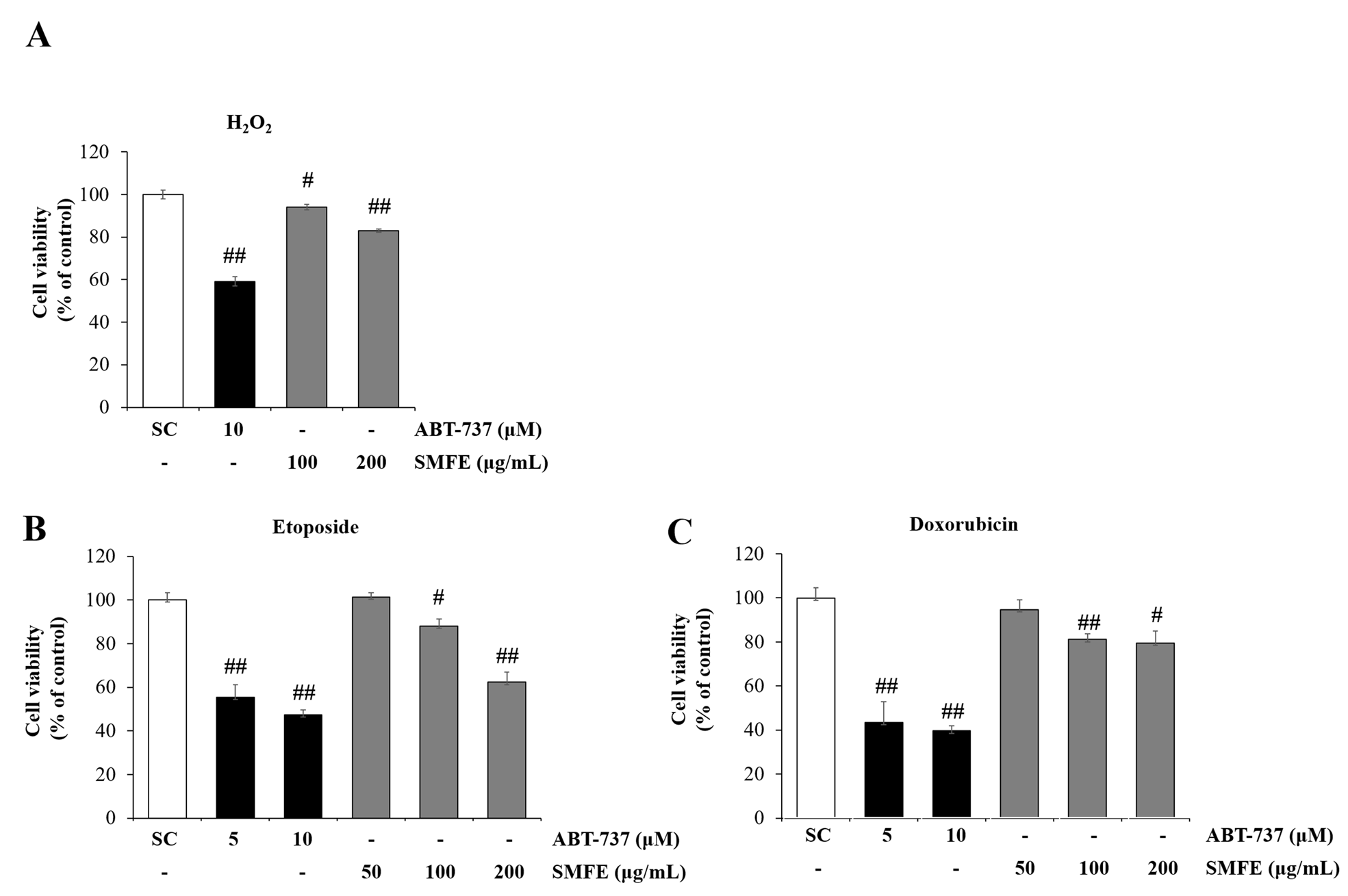

Supplement: S2 Fig — Senolytic effect of SMFE in senescence model caused by external stimulation, hydrogen peroxide (A), etoposide (B), and doxorubicin (C). Data are represented as mean ± SEM of three independent assays. *p < 0.05; **p < 0.01 compared to young cell control, #p < 0.05; ##p < 0.01 compared to senescent cell control. SC, senescent cell control. (TIF) [file pone.0260545.s002.tif]

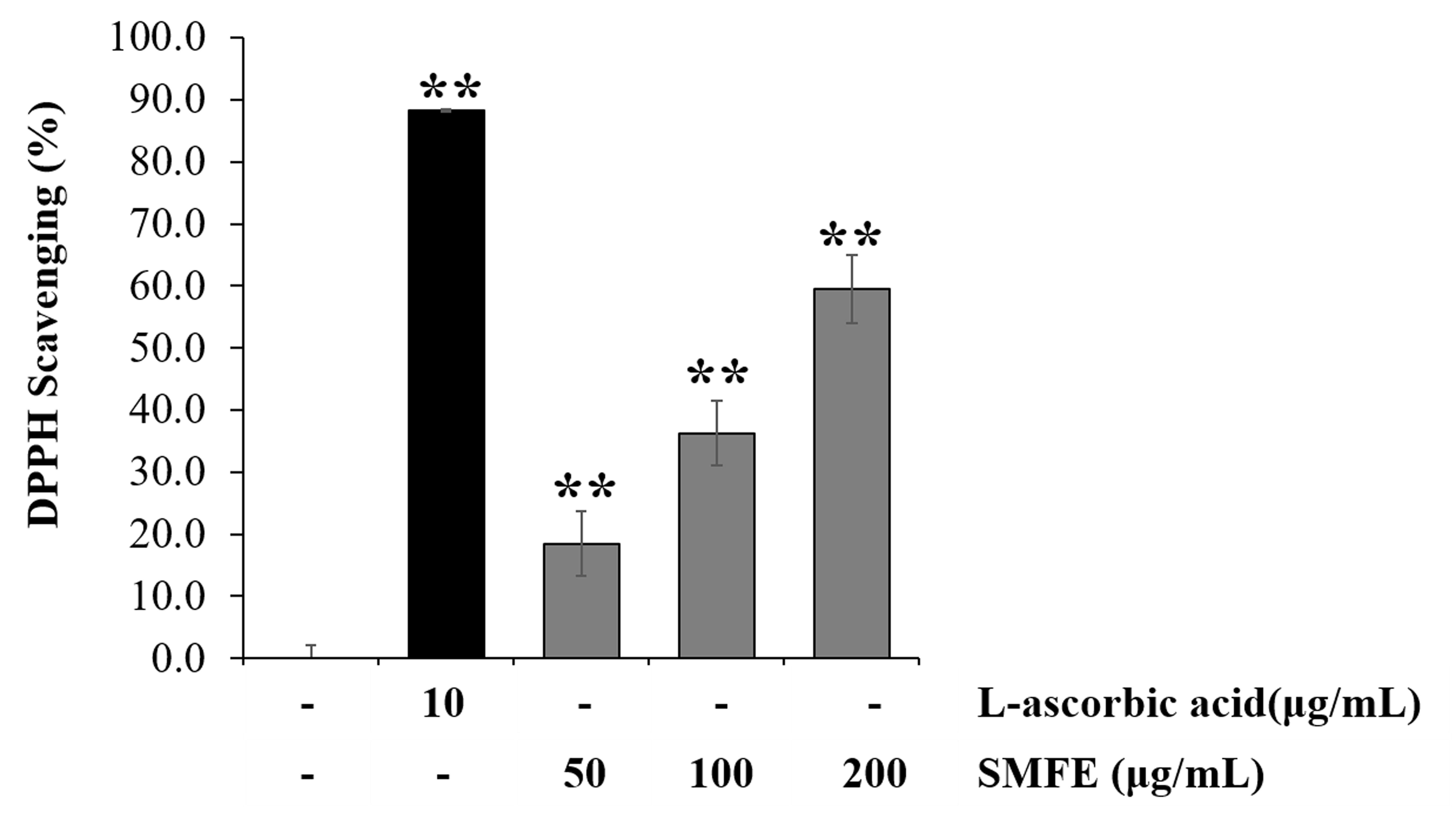

Supplement: S4 Fig — Data are represented as mean ± SEM of three independent assays. *p < 0.05; **p < 0.01 compared to control. (TIF) [file pone.0260545.s004.tif]
